# Nanoscale Features of Gambogic Acid Induced ROS-Dependent Apoptosis in Esophageal Cancer Cells Imaged by Atomic Force Microscopy

**DOI:** 10.1155/2022/1422185

**Published:** 2022-07-22

**Authors:** Jianxin Liu, Shuhao Fan, Yinhong Xiang, Jiaojiao Xia, Hua Jin, Jun-fa Xu, Fen Yang, Jiye Cai, Jiang Pi

**Affiliations:** ^1^Hunan Provincial Key Laboratory of Dong Medicine, Hunan Province Key Laboratory for Antibody-Based Drug and Intelligent Delivery System, Hunan Provincial Key Laboratory for Synthetic Biology of Traditional Chinese Medicine, School of Pharmaceutical Sciences, Hunan University of Medicine, Huaihua, China; ^2^Institute of Laboratory Medicine, Guangdong Provincial Key Laboratory of Medical Molecular Diagnostics, School of Medical Technology, The First Dongguan Affiliated Hospital, Guangdong Medical University, Dongguan, China; ^3^School of Basic Medical Sciences, Hunan University of Medicine, Huaihua, China; ^4^Department of Chemistry, Jinan University, Guangzhou, China

## Abstract

Gambogic acid (GA), a kind of polyprenylated xanthone derived from Garcinia hanburyi tree, has showed spectrum anticancer effects both in vitro and in vivo with low toxicity. However, up to now, there is little information about the effects of GA on esophageal cancer. In this study, we aim to test the anticancer effects of GA on esophageal cancer EC9706 cells. We established a nanoscale imaging method based on AFM to evaluate the reactive oxygen species- (ROS-) mediated anticancer effects of GA on esophageal cancer regarding the morphological and ultrastructural changes of esophageal cancer cells. The obtained results demonstrated that GA could inhibit cell proliferation, induce apoptosis, induce cell cycle arrest, and induce mitochondria membrane potential disruption in a ROS-dependent way. And using AFM imaging, we also found that GA could induce the damage of cellular morphology and increase of membrane height distribution and membrane roughness in EC9706 cells, which could be reversed by the removal of GA-induced excessive intracellular ROS. Our results not only demonstrated the anticancer effects of GA on EC9706 cells in ROS-dependent mechanism but also strongly suggested AFM as a powerful tool for the detection of ROS-mediated cancer cell apoptosis on the basis of imaging.

## 1. Introduction

In recent decades, the cases of cancer are still increasing all over the world, which dramatically threaten the life of humans. Esophageal cancer is a public health problem in many countries, especially in emerging and developing countries. This cancer happens in the esophagus—the food pipe that runs between the throat and the stomach. Esophageal cancer might be induced by tobacco, alcohol, very hot drinks, a poor diet, obesity, and acid reflux [1, 2]. Esophageal cancer can cause the difficulty or pain in swallowing, enlarged lymph nodes around the collarbone, dry cough, possibly coughing up, vomiting blood, weight loss, and finally apoptosis, seriously affecting the quality of human life. Up to now, the therapy approaches for esophageal cancer mainly include esophagectomy, chemotherapy, and radiotherapy. But all these approaches have strong side effects on human health, therefore making the search of new treatments for esophageal cancer as one of the most urgent issues for cancer therapy.

Gambogic acid (GA; C_38_H_44_O_8_, MW: 628.76), a polyprenylated xanthone and a widely used coloring agent, is the main active ingredient of gamboges secreted from the Garcinia hanburyi tree ([3, 4], which mainly grows in Southeast Asia. It is an active compound derived from traditional Chinese medicine; GA is used for various human condition treatments for hundreds of years. In recent decades, GA is widely reported to show spectrum anticancer effects both in vitro and in vivo with low toxic side effects, showing potential use for cancer treatment clinically [5–8]. And additionally, GA has been approved by the Chinese FDA for the treatment of solid cancers in Phase II clinical trials. It has been reported that GA could induce cancer cell apoptosis by inducing the over-production of reactive oxygen species (ROS) in cancer cells, and the removal of ROS could therefore reverse GA-induced cancer cell apoptosis [9–11]. GA is also found to enhance the radiosensitivity of human esophageal cancer cells [12]. However, the exact anticancer effects of GA on esophageal cancer still require further investigation.

Atomic force microscopy (AFM) has been developed into a powerful nanotechnique for biological sample detection, which permits the observation of biological samples ranging from a single molecule to whole cells because of its nanometer spatial resolution. In recent years, taking the advantages of high-resolution imaging, the application of AFM has been extended for cell detection [13–15]. Based on its high sensitivity in studying cellular samples, AFM is widely used for the pharmacological effect investigations of drugs *in vitro* [16–18]. However, there are still no studies investigating the effects of GA on cancer cells by AFM.

In the present work, we conducted a battery of evaluations on the basis of EC9706 cells to demonstrate the anticancer effects of GA on esophageal cancer cells. GA was found to inhibit the proliferation of EC9706 cells by inducing ROS production, and the removal of GA-induced excessive ROS by N-acetyl-L-cysteine (NAC) could reverse GA-inhibited EC9706 cell proliferation. GA could also induce apoptosis, cell cycle arrest, and mitochondrial membrane potential disruption of EC9706 cells, and the removal of GA-induced excessive ROS could reverse all these effects. Notably, we found that GA could induce morphological damage and membrane ultrastructure changes of EC96706 cells using AFM, which could be reversed by the removal of GA-induced excessive ROS.

## 2. Materials and Methods

### 2.1. Materials

Gambogic acid (98%, HPLC) was obtained from YiJi biotechnology (China). Fetal bovine serum (FBS), penicillin/streptomycin, Dulbecco's modified eagle medium (DMEM), and trypsin kit were obtained from Gibco (USA). Paraformaldehyde was purchased from Sigma (USA). Annexin V-FITC/PI apoptosis detection kit was obtained from BD Biosciences (USA). 3-(4,5)-Dimethylthiazol(-z-y1)-3,5-diphenyt-etrazoliumromide (MTT), N-acetyl-L-cysteine (NAC), rhodamin 123, DCFH-DA (2′,7′-dichlorodihydrofluorescein diacetate) reactive oxygen species assay kit, and cell cycle analysis kits were purchased from Beyotime Institute of Biotechnology (China).

### 2.2. Cell Culture

Human esophageal cancer EC9706 cells were cultured with DMEM supplemented with 10% FBS, 100 U/mL penicillin, and 100 g/mL streptomycin in a humidified atmosphere of 5% CO_2_ at 37°C.

### 2.3. MTT Assay

MTT (3-(4, 5)-dimethylthiazol(-z-y1)-3,5-diphenyt-etrazoliumromide) assay was used to test the effects of GA on the viability of EC9706 cells. The cells were seeded into 96 well plates with a density of 5 × 10^3^ cells/well for 24 h and incubated with different concentrations of GA for 24 h. To remove GA-induced excessive ROS, cells were pretreated with 5 mM NAC for 1 h and then treated with GA for 24 h. After GA treatment, MTT reagent (10 *μ*L, 5 mg/mL) was then added into each well for 4 h incubation, the medium was removed, and the cells were suspended in 150 *μ*L DMSO to incubate for 10 min. A spectrophotometer (TECAN, Switzer-land) was used to test absorbance at 570 nm with a reference wavelength at 650 nm.

### 2.4. ROS Assay

A DCFH-DA-based reactive oxygen species assay kit was used to determine the intracellular ROS level of EC9706 cells. The cells were seeded into 6-well plates with a density of 1 × 10^5^ cells/well for 24 h and incubated with different concentration of GA for 3 h. To remove GA-induced excessive ROS, cells were pretreated with 5 mM NAC for 1 h and then treated with GA for 3 h. After GA treatment, cells were harvested, washed triple with PBS, and incubated with DCFH-DA solution for 30 min in the dark at 37°C. Flow cytometry (BD, USA) was used to detect the intracellular ROS level after the cells were collected and washed twice with PBS.

### 2.5. Apoptosis and Necrosis Assay

Annexin V-FITC/PI apoptosis detection kit was used to detect the effects of GA on the apoptosis EC9706 cells. The cells were seeded into 6-well plates with a density of 1 × 10^5^ cells/well for 24 h and incubated with different concentrations of GA for 24 h. To remove GA-induced excessive ROS, cells were pretreated with 5 mM NAC for 1 h and then treated with GA for 24 h. After being incubated with GA, EC9706 cells were harvested, washed three times with PBS, suspended in Annexin V binding buffer, and incubated with Annexin V-FITC and PI for 5 min at room temperature in the dark. Then, the samples were immediately analyzed by flow cytometry (BD, USA).

### 2.6. Mitochondrial Membrane Potential Analysis

Rhodamine 123 was used as a fluorescence to determine the alterations of mitochondrial membrane potential (MMP) of EC9706 cells upon GA treatment. The cells were seeded into 6-well plates with a density of 1 × 10^5^ cells/well for 24 h and incubated with different concentrations of GA for 24 h. To remove GA-induced excessive ROS, cells were pretreated with 5 mM NAC for 1 h and then treated with GA for 24 h. The harvested and washed EC9706 cells were incubated with rhodamine 123 for 60 min in the dark at 37°C. Flow cytometry (BD, USA) was used to detect the fluorescence signal of rhodamine 123 after the cells were collected and washed twice with PBS.

### 2.7. AFM Sample Preparation and AFM Measurements

To prepare AFM imaging samples, EC9706 cells were cultured at a density of 5 × 10^4^ cells/well on glass coverslips in a six-well plate. After overnight incubation, EC9706 cells were treated with GA for 24 h. To remove GA-induced excessive ROS, cells were pretreated with 5 mM NAC for 1 h and then treated with GA for 24 h. After GA treatment, cells were washed triple with PBS, fixed with 4% paraformaldehyde solution for 10 min, washed triple with distilled water, and dried in air for morphology imaging in air.

AFM was used to determine the morphological and ultrastructural (BioScope Catalyst, Bruker, Germany) changes of EC9706 cells induced by GA treatment. Silicon nitride tips were used in the experiments; they were irradiated by ultraviolet irradiation to remove the impurity on tip surface. The curvature radius of the pyramid AFM tips with a triangle cantilever (SiNi-100 tips, BudgetSensors, Bulgaria) used for morphology imaging was 10 nm, and the spring constant of tips determined by thermal tuning was 0.86 ± 0.19 N/m. The morphological and ultrastructural images of EC9706 cells were obtained in air at room temperature in a contact mode with 256 × 256 pixels and scan rate of 5 min per image. The ultrastructure of EC9706 cells was obtained in the areas surrounding the nuclei, and the topographical image processing and data analysis were performed using the instrument equipped Nanoscope Analysis software.

### 2.8. Statistical Analysis

All data presented are expressed as mean ± S.E.M. and representative of three independent experiments in independent samples. Statistical analysis was performed using Student's *t*-test, and *p* < 0.05 was regarded as statistically significant.

## 3. Results and Discussion

### 3.1. Effects of GA on EC9706 Cell Proliferation

**Figure 1:**
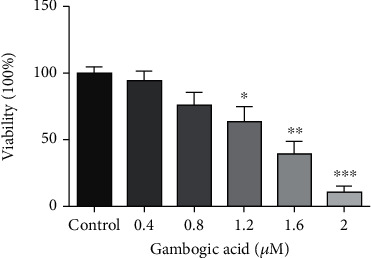
Effects of gambogic acid on the viability of EC9706 cells after 24 h treatment. *n* = 3; ^∗^*p* < 0.05,  ^∗∗^*p* < 0.01, and^∗∗∗^*p* < 0.001.

GA has been reported to show strong proliferation inhibition effects on cancer cells, including lymphoma cells [19], breast cancer cells [20], colorectal cancer cells [21], glioma cells [22], and bladder cancer cells [23]. Yang et al. also reported the proliferation inhibition effects of GA on esophageal cancer TE13 cells [12]. Here, we determine the effects of GA on the proliferation of esophageal cancer EC9706 cells by MTT assay, which was based on the cleavage of the tetrazolium ring of MTT (3-(4,5-dimethylthazolk-2-yl)-2,5-diphenyl tetrazolium bromide) by dehydrogenases in active mitochondria of living cells as an estimate of viable cell number. As shown in Figure 1, after 24 h GA treatment, the viability of EC9706 cells decreased from 100.00 ± 4.30% for control cells to 94.10 ± 7.37%, 75.72 ± 9.55%, 63.04 ± 11.70%, 39.03 ± 9.69%, and 10.47 ± 4.72% for 0.4 *μ*M, 0.8 *μ*M, 1.2 *μ*M, 1.6 *μ*M, and 2 *μ*M GA-treated EC9706 cells, respectively. These results demonstrated the strong inhibition effects of GA on esophageal cancer EC9706 cells.

### 3.2. Effects of GA on Intracellular ROS Level in EC9706 Cells

**Figure 2:**
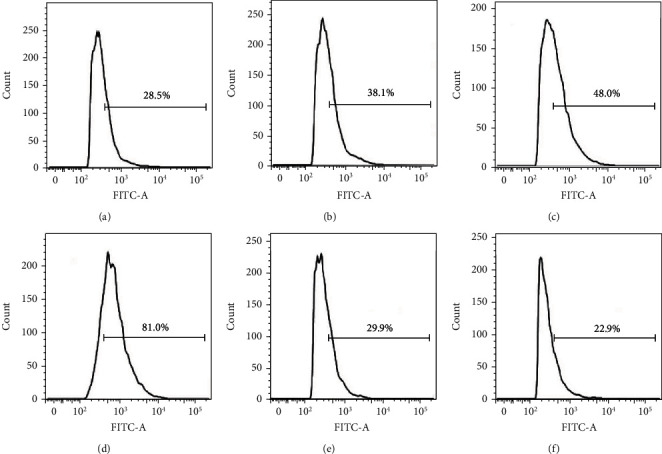
Effects of gambogic acid on ROS production in EC9706 cells after 3 h treatment. Intracellular ROS level of (a) control, (b) 0.4 *μ*M gambogic acid-treated, (c) 1.2 *μ*M gambogic acid-treated, (d) 2 *μ*M gambogic acid-treated, (e) 5 mM NAC+2 *μ*M gambogic acid-treated, and (f) 5 mM NAC-treated EC9706 cells. EC9706 cells in (e, f) were pretreated with 5 mM NAC for 1 h and then treated with or without gambogic acid for 3 h to determine the effects of ROS scavenger-NAC on gambogic acid-induced ROS production in EC9706 cells.

Some previous works focused on the investigation of anticancer activity of GA implied that the inhibition effects of GA on cancer cells were always associated with the increase of intracellular ROS level [9–11]. To further know the anticancer mechanism of GA in EC9706 cells, we further determined the effects of GA on the intracellular ROS level by DCFH-DA-based ROS assay kit. After diffusion of DCFH-DA into the cell, it can be deacetylated by cellular esterases to a nonfluorescent compound, which is later oxidized by ROS into 2′,7′-dichlorofluorescein (DCF). DCF is highly fluorescent and can be detected by flow cytometry with excitation/emission at 485 nm/535 nm. As shown in Figure 2, the average intracellular ROS level increased from 28.5% to 38.1%, 48%, and 81% after 0.4 *μ*M, 1.2 *μ*M, and 2 *μ*M GA treatment, respectively. These results suggested that GA could dramatically increase the intracellular ROS level in EC9706 cells. With the pretreatment of ROS scavenger-NAC for 1 h, the intracellular ROS level in 2 *μ*M GA-treated EC9706 cells was 29.9%, and the intracellular ROS level in EC9706 cells without GA treatment was 22.9%. These results indicated the strong ability of NAC to remove GA-induced excessive ROS in EC9706 cells.

### 3.3. Effects of NAC on GA Increased Intracellular ROS Level in EC9706 Cells

**Figure 3:**
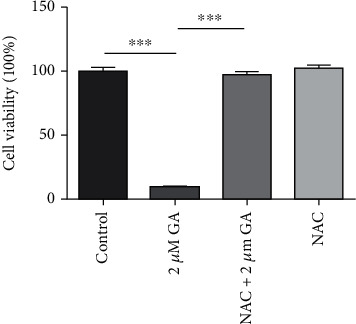
Effects of ROS scavenger-NAC on gambogic acid inhibited viability of EC9706 cells. EC9706 cells were pretreated with 5 mM NAC for 1 h and then treated with or without gambogic acid for 24 h. *n* = 3; ^∗∗∗^*p* < 0.001.

ROS have been widely reported to play critical roles in the anticancer mechanisms of anticancer drugs [24]. GA has also been found to induce ROS in cancer cells [25], which highlight the potential roles of ROS in GA-inhibited esophageal cancer. To determine the roles of ROS in the anticancer activity of GA, we also detected the effects of NAC on GA inhibited EC9706 cell proliferation. As indicated by Figure 3, with 24 h 2 *μ*M GA treatment, the viability of EC9706 cells decreased from 100.00 ± 3.34% to 8.96 ± 0.18%. However, with NAC pretreatment for 1 h, the viability of 2 *μ*M GA-treated EC9706 cells reversed to 96.97 ± 2.87%. The treatment only with NAC showed no significant effects on EC9706 cells, which showed an average viability of 102.30 ± 2.50%. These results strongly suggested that GA could inhibit EC9706 cell proliferation in a ROS-dependent way. Previous works have also reported the ROS-dependent inhibition effects of GA on cancer cell proliferation and the pretreatment with NAC could reverse GA inhibited colorectal cancer cells [11] and myeloma cells [10]. Our results further suggested that GA might inhibit cancer cell proliferation via a ROS-dependent way.

### 3.4. Effects of GA on EC9706 Cell Apoptosis and Necrosis

**Figure 4:**
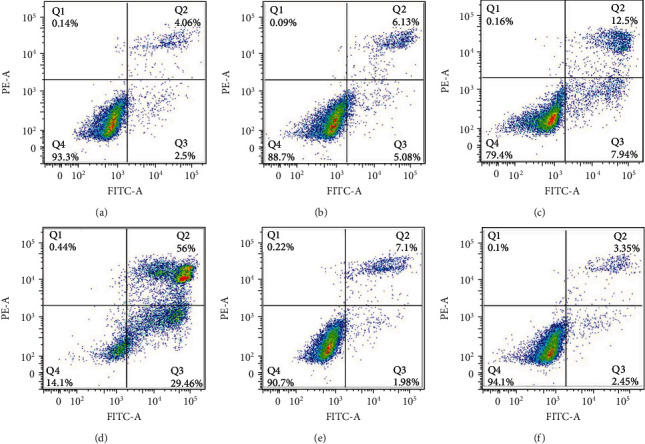
Effects of gambogic acid on apoptosis of EC9706 cells after 24 h treatment. Intracellular ROS level of (a) control, (b) 0.4 *μ*M gambogic acid-treated, (c) 1.2 *μ*M gambogic acid-treated, (d) 2 *μ*M gambogic acid-treated, (e) 5 mM NAC+2 *μ*M gambogic acid-treated, and (f) 5 mM NAC-treated EC9706 cells. EC9706 cells in (e, f) were pretreated with 5 mM NAC for 1 h and then treated with or without gambogic acid for 24 h to determine the effects of ROS scavenger-NAC on gambogic acid induced apoptosis in EC9706 cells.

Anticancer drugs always showed strong ability to induce apoptosis and necrosis in cancer cells. To further understand GA-induced EC9706 cell apoptosis, we determined the apoptosis and necrosis rate of CE9706 cells upon GA treatment by Annexin V-FITC (fluorescein isothiocyanate)/PI (Propidium Iodide) assay. Annexin V is an anticoagulant protein that preferentially binds negatively charged phospholipids. Early apoptosis is characterized by the eversion of cell membrane, which result in the phosphatidylserine exposure for Annexin V binding. Late apoptosis and necrosis are characterized by the broken of cell membrane, which result in the phosphatidylserine exposure for Annexin V binding and the binding of PI with the intracellular DNA. Thus, Annexin V/PI staining assay can be used to distinguish early apoptosis from late apoptosis and necrosis by flow cytometry analysis. For flow cytometry result analysis as shown in Figure 4, Annexin V-negative and PI-negative cells (Q4) are normal cells without apoptosis and necrosis, Annexin V-positive and PI-negative cells (Q3) are apoptotic cells suffering from early apoptosis, and Annexin V-positive and PI-positive cells (Q2) are necrotic cells suffering from necrosis and late apoptosis. As shown in Figure 4, the living cells (Q4) for the control group decreased from 93.3% to 88.7%, 79.4%, and 14.1% after 0.4 *μ*M, 1.2 *μ*M, and 2 *μ*M GA treatment, respectively. However, with NAC pretreatment for 1 h, the living cells of 2 *μ*M GA-treated EC9706 cells reversed to 89.4%, and the living cells of EC9706 cells without GA treatment changed to 93.0%. And the early apoptotic cells (Q3) increased from 2.45% to 5.08%, 7.94%, and 29.46% after 0.4 *μ*M, 1.2 *μ*M, and 2 *μ*M GA treatment, respectively.

With NAC pretreatment for 1 h, the 2 *μ*M GA-treated cells suffering from early apoptosis were reversed to 1.98%, while the cells without GA treatment showed early apoptosis of 2.45%. Additionally, the cells suffering from late apoptosis and necrosis increased from 4.06% for control cells to 6.13%, 12.5%, and 56% for 0.4 *μ*M, 1.2 *μ*M, and 2 *μ*M GA-treated cells, respectively. Similarly, with NAC pretreatment for 1 h, the late apoptosis and necrosis cells of 2 *μ*M GA-treated EC9706 cells reversed to 7.1%. And the apoptosis and necrosis cells of NAC-pretreated EC9706 cells without GA treatment (NAC control) changed to 3.35%. These results demonstrated that GA could induce EC9706 cell apoptosis by the induction of apoptosis and necrosis in a ROS-dependent way, and the removal of GA-induced excessive ROS by NAC could reverse GA-induced EC9706 cell apoptosis and necrosis.

### 3.5. Effects of GA on EC9706 Cell Cycle Arrest

**Figure 5:**
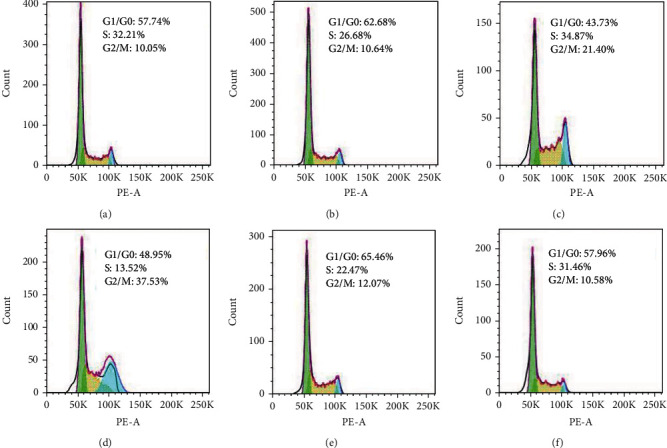
Effects of gambogic acid on cell cycle distribution of EC9706 cells after 24 h treatment. Intracellular ROS level of (a) control, (b) 0.4 *μ*M gambogic acid-treated, (c) 1.2 *μ*M gambogic acid-treated, (d) 2 *μ*M gambogic acid-treated, (e) 5 mM NAC+2 *μ*M gambogic acid-treated, and (f) 5 mM NAC-treated EC9706 cells. EC9706 cells in (e, f) were pretreated with 5 mM NAC for 1 h and then treated with or without gambogic acid for 24 h to determine the effects of ROS scavenger-NAC on gambogic acid induced cell cycle arrest in EC9706 cells.

We further investigated the effects of GA on the cell cycle distribution of EC9706 cells by PI staining. Due to the different DNA contents in the three interphase stages of the cell cycle, the DNA binding fluorescent PI can therefore be used to identify the proportion of cells that are in one of the three interphase stages of the cell cycle by flow cytometry analysis. In theory, cells in G2/M phase (G2 phase and M phase in cell cycle) would show double fluorescence signals than the G0/G1 phase (G0 phase and G1 phase in cell cycle), and the S phase would show fluorescence signals ranging from the G0/G1 phase to G2/M phase. The results (Figure 5) implied that the cells at G2/M phase increased from 10.05% to 10.64%, 21.40%, and 37.53% after 0.4 *μ*M, 1.2 *μ*M, and 2 *μ*M GA treatment, respectively. But with NAC pretreatment for 1 h, the 2 *μ*M GA-treated cells at G2/M phase reversed to 12.07%. And the NAC-pretreated cells without GA treatment at G2/M phase changed to 10.58%. These results demonstrated that GA could induce EC9706 cell cycle arrest at G2/M phase in ROS-dependent way, and the removal of GA-induced excessive ROS by NAC could reverse GA-induced EC9706 cell cycle arrest.

### 3.6. Effects of GA on Mitochondria Membrane Potential of EC9706 Cells

**Figure 6:**
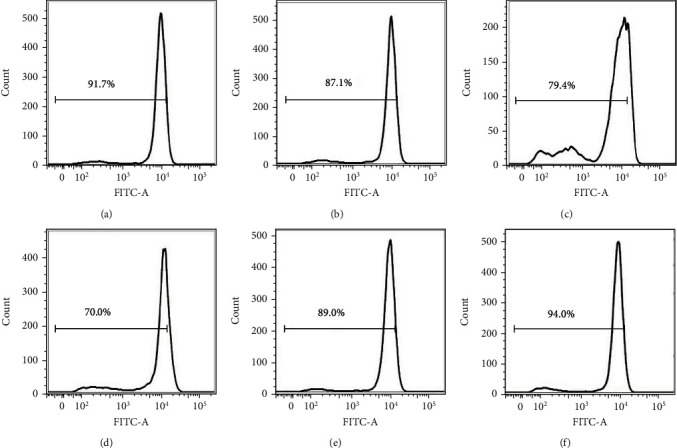
Effects of gambogic acid on mitochondrial membrane potential of EC9706 cells after 24 h treatment. Intracellular ROS level of (a) control, (b) 0.4 *μ*M gambogic acid-treated, (c) 1.2 *μ*M gambogic acid-treated, (d) 2 *μ*M gambogic acid-treated, (e) 5 mM NAC+2 *μ*M gambogic acid-treated, and (f) 5 mM NAC-treated EC9706 cells. EC9706 cells in (e, f) were pretreated with 5 mM NAC for 1 h and then treated with or without gambogic acid for 24 h to determine the effects of ROS scavenger-NAC on gambogic acid induced mitochondrial membrane potential disruption in EC9706 cells.

The disruption of mitochondrial membrane potential (MMP) of cancer cells is a common feature for anticancer drug-induced cancer cell apoptosis, which is also associated with the induction of ROS [26, 27]. To determine if the dysfunction of mitochondria was also involved in GA-induced EC9706 cell apoptosis, we examined the MMP in response to GA exposure by flow cytometry using rhodamine 123 as a fluorescence probe. Rhodamine 123 is a cationic fluorescent dye that is used to specifically label respiring mitochondria. The dye distributes according to the negative membrane potential across the mitochondrial inner membrane, and the loss of potential will result in the loss of the dye and, therefore, the decrease fluorescence intensity that can be detected by flow cytometry. The results also demonstrated the dose-dependent decrease of MMP in EC9706 cells after GA treatment. As shown in Figure 6, the relative MMP of control EC9706 cells decreased from 91.7% to 87.1%, 79.4%, and 70.0% after 0.4 *μ*M, 1.2 *μ*M, and 2 *μ*M GA treatment, respectively. With NAC pretreatment for 1 h, the MMP of 2 *μ*M GA-treated cells reversed to 89.0%, and the MMP of cells without GA treatment changed to 94.0%. These results strongly suggested that GA could induce the disruption of MMP in EC9706 cells in a ROS-dependent manner, and the removal of GA-induced excessive ROS by NAC could reverse GA-induced EC9706 cell MMP disruption.

### 3.7. Effects of GA on Morphology of EC9706 Cells

Cell morphology is closely related to the physiological status and function of cells. The resolution that AFM possessed could provide the convenience for cell morphology study by directly imaging of precise membrane ultrastructure changes [28, 29]. Here, for the first time, we applied AFM to detect the effects of GA on the morphology of EC9706 cells. Fixation of the cell samples is necessary because higher structures of living cells were frequently too pliable for reproducible AFM scanning [30]. Thus, the EC9706 cell samples were fixed to increase reproducibility imaging by AFM despite the possibility of artefacts to be added due to this fixing process.

**Figure 7:**
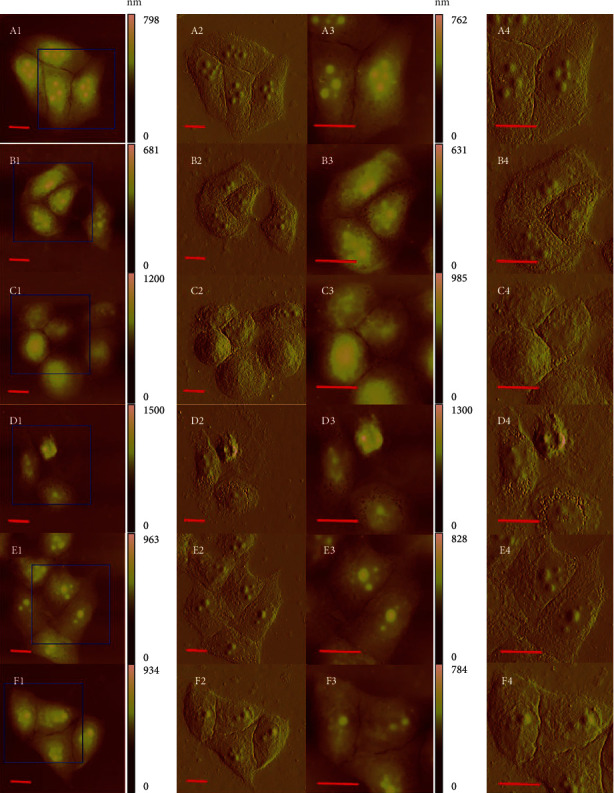
Effects of gambogic acid on cell morphology of EC9706 cells after 24 h treatment. AFM morphology imaging of (a) control, (b) 0.4 *μ*M gambogic acid-treated, (c) 1.2 *μ*M gambogic acid-treated, (d) 2 *μ*M gambogic acid-treated, (e) 5 mM NAC+2 *μ*M gambogic acid-treated, and (f) 5 mM NAC-treated EC9706 cells. (A1-F1) Topography images and (A2-F2) their corresponding deflection error images. (A3-F3) Enlarged topography images as indicated by the blue frames in (A1-F1) and (A4-F4) their corresponding deflection error images. Scale bar: 20 *μ*m. EC9706 cells in (e, f) were pretreated with 5 mM NAC for 1 h and then treated with or without gambogic acid for 24 h to determine the effects of ROS scavenger-NAC on gambogic acid induced morphological damage in EC9706 cells by AFM imaging in air with contact mode.

As shown in Figure 7(a), control EC9706 cells showed irregular shapes and the cells were in close contact with one another. After treated with 0.4 *μ*M GA for 24 h, there were no remarkable changes in the morphology of EC9706 cells (Figure 7(b)). After 24 h 1.2 *μ*M GA treatment, the irregular shaped cells seemed to show oval shapes (Figure 7(c)). And for 2 *μ*M GA-treated EC9706 cells, morphological characteristics of cell apoptosis could be observed, such as the shrinking of cell bodies, the condensation of cytoplasm, and the condensation and fragmentation of nucleus (Figure 7(d)). These results demonstrated that GA treatment could induce morphological damage of EC9706 cells. To determine the roles of ROS in GA-induced cell morphological damage, cells were pretreated with NAC for 1 h. With NAC pretreatment, the morphology of 2 *μ*M GA-treated EC9706 cells was found to be similar with that of control EC9706 cells (Figure 7(e)). And the NAC pretreatment also showed no significant effects on the morphology of EC9706 cells (Figure 7(f)). We also showed the 3-D images of these AFM images in the supplementary materials (Figure 1).

These AFM images with an imaging size of 100 *μ*m × 100 *μ*m (Figure 7 A1-F1) and 60 *μ*m × 60 *μ*m (Figure 7 A3-F3) suggested that GA could induce morphological damages of EC9706 cells in a ROS-dependent way and these morphological damages could also be reversed by NAC pretreatment, indicating that AFM can be used as a qualitative nanotool to determine ROS-mediated cell morphological changes in cancer cell apoptosis. Our results further proved the ability of AFM to detect ROS-mediated cancer cell apoptosis.

### 3.8. Effects of GA on Membrane Ultrastructure of EC9706 Cells

Although the morphology of EC9706 cells determined by AFM could be used to describe GA-induced EC9706 cell apoptosis in a ROS-dependent way, it is still difficult to be used as a quantitative tool for cancer cell apoptosis analysis. As cell apoptosis is always associated with the changes of cell membrane components, such as lipid raft, membrane proteins, and phospholipid [31, 32], AFM-detected cell membrane ultrastructure could be widely used to evaluate cancer cell apoptosis [16, 17, 28, 33–35]. Based on the AFM images, quantitative parameters describing the property of cell membrane ultrastructure could be extracted to clarify the changes of cell membrane ultrastructure during cell apoptosis.

**Figure 8:**
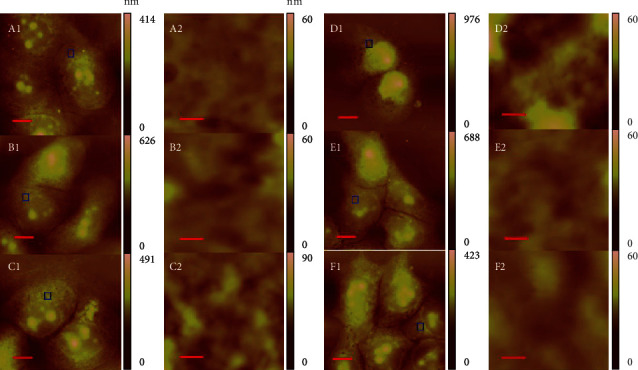
Effects of gambogic acid on cell membrane ultrastructure of EC9706 cells after 24 h treatment. AFM morphology and membrane ultrastructure images of (a) control, (b) 0.4 *μ*M gambogic acid-treated, (c) 1.2 *μ*M gambogic acid-treated, (d) 2 *μ*M gambogic acid-treated, (e) 5 mM NAC+2 *μ*M gambogic acid-treated, and (f) 5 mM NAC-treated EC9706 cells. (A1-F1) Topography images of EC9706 cells, scale bar: 10 *μ*m. (A2-F2) Enlarged cell membrane ultrastructure images of EC9706 cells as indicated by the blue frames in (A1-F1); scale bar: 400 nm. EC9706 cells in (e, f) were pretreated with 5 mM NAC for 1 h and then treated with or without gambogic acid for 24 h to determine the effects of ROS scavenger-NAC on gambogic acid induced membrane ultrastructure changes in EC9706 cells by AFM imaging in air with contact mode.

Therefore, AFM image analysis was performed to investigate the more detailed morphological changes of GA-induced EC9706 cell apoptosis. To avoid the height and roughness variability across different parts of cells, we chose to investigate the ultrastructure of EC9706 cells in the areas surrounding the nuclei and far from the edge of cells. As shown in Figure 8 A2, control EC9706 cells showed relative smooth membrane ultrastructure and the height distribution analyzed by the cell membrane ultrastructure showed average diameter of 20 nm (Supplementary materials. Fig. 2). In the 0.4 *μ*M GA-treated group, the ultrastructure of EC9706 cells showed more significant hole-like structures (Figure 8 B2), and the height distribution analyzed by the cell membrane ultrastructure showed average diameter of 30 nm (Supplementary materials. Fig. 2). The membrane ultrastructures of 1.2 *μ*M GA-treated EC9706 cells (Figure 8 C2) and 2 *μ*M GA-treated EC9706 cells (Figure 8 D2) both showed more remarkable hole-like structures, indicating the damage of cell membrane induced by GA. And the height distribution analyzed by the cell membrane ultrastructure showed average diameter of 45 nm and 55 nm for 1.2 *μ*M GA-treated EC9706 cells and 2 *μ*M GA-treated EC9706 cells (Supplementary materials. Fig. 2), respectively. NAC was also added for 1 h pretreatment to determine the effects of ROS on GA-induced EC9706 cell membrane ultrastructure changes. The hole-like structures in 2 *μ*M GA-treated EC9706 cells could be reversed by NAC pretreatment resulting in a similar structure as the control cells (Figure 8 E2), and the height distribution analyzed by the cell membrane ultrastructure was also reversed to 25 nm (Supplementary materials. Fig. 2). Additionally, just NAC pretreatment had no significant effects on the cell membrane ultrastructure of EC9706 cells and the height distribution analyzed by the cell membrane ultrastructure showed an average diameter of 20 nm.

**Figure 9:**
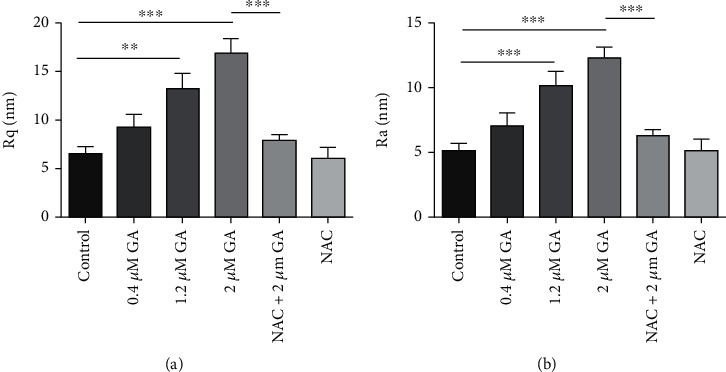
Statistical analysis of the effects of gambogic acid on membrane ultrastructure of EC9706 cells after 24 h treatment and the effects of ROS scavenger-NAC on gambogic acid induced EC9706 cell membrane ultrastructural changes determined by AFM. (a) Root mean square roughness (Rq) and (b) average roughness (Ra) analyzed from 2 × 2 *μ*m frame ultrastructure images of EC9706 cells. *n* = 10; ^∗∗^*p* < 0.01 and^∗∗∗^*p* < 0.001.

Then, by studying 10 different cell membrane ultrastructure images in each group, we found that the height distribution of control EC9706 cells was 20.37 ± 3.70 nm, which increased to 28.81 ± 5.03 nm, 43.39 ± 6.45 nm, and 55.93 ± 7.44 nm after 0.4 *μ*M, 1.2 *μ*M, and 2 *μ*M GA treatment, respectively (Supplementary materials. Figure 2). And with NAC pretreatment for 1 h, height distribution of 2 *μ*M GA-treated EC9706 cells reversed to 26.94 ± 4.15 nm and the height distribution of 2 *μ*M GA-treated EC9706 cells changed to 19.42 ± 2.97 nm (Supplementary materials. Fig. 2). Roughness is another important parameter that describes the changes of membrane topography in cells with and without drug treatment [16, 28, 29]. To further clarify the effects of GA on the membrane ultrastructure of EC9706 cells, the roughness of the obtained membrane ultrastructure images was analyzed, which are presented in Figures 9(a) and 9(b). The root mean square roughness (Rq) of control EC9706 cells was 6.51 ± 0.73 nm, which increased to 9.22 ± 1.35 nm, 13.22 ± 1.58 nm, and 16.78 ± 1.61 nm after 0.4 *μ*M, 1.2 *μ*M, and 2 *μ*M GA treatment, respectively. NAC pretreatment, which showed Rq value of 6.03 ± 1.15 nm in control EC9706 cells was found to reverse the Rq values of 2 *μ*M GA-treated EC9706 cells to 7.88 ± 0.60 nm. Additionally, the average roughness (Ra) of EC9706 cells changed from 5.11 ± 0.59 nm for control cells to 7.05 ± 0.98 nm, 10.13 ± 1.12 nm, and 12.24 ± 0.89 nm for 0.4 *μ*M, 1.2 *μ*M, and 2 *μ*M GA-treated EC9706 cells. And with NAC pretreatment, the Ra value of control EC9706 cells was 5.12 ± 0.88 nm. And the Ra value of 2 *μ*M GA-treated EC9706 cells was reversed to 6.23 ± 0.50 nm with NAC pretreatment for 1 h. These results strongly suggested that GA could induce the increase of membrane height distribution and roughness in EC9706 cells with a ROS-dependent manner, and by removing GA-induced excessive ROS by NAC, these effects could be reversed.

The ultrastructure of cell membrane is closely related to the metastasis state of cells, such as motility, adhesion, and intracellular contact, therefore making it a good indicator for the health or abnormal status of cells. The increased height distribution on cell surface might be attributed to the formation of holes on surface and the aggregation of membrane biomolecules [35]. These results indicated that GA-induced EC9706 cell apoptosis might be partially due to the aggregation of membrane biomolecules in a ROS-dependent way, and the removal of ROS by NAC could reverse this effects. The hole-like structures on cell membrane are always regarded to lead a rougher surface of cells [34]. GA was found to induce hole-like structures in EC9706 cell surface by our AFM imaging, which also induced the increase of cell membrane roughness in EC9706 cells. And the removal of GA induced ROS caused the reverse of GA induced hole-like structures in EC9706 cells, which therefore decreased the roughness in the membrane of EC9706 cells.

In this work, we mainly determined the morphological and surface ultrastructural of EC9706 cells upon GA treatment; these results not only indicated the anticancer effects of GA on esophageal cancer cells in a ROS-dependent way but also further proved the potential use of AFM for the study of ROS-mediated cancer cell apoptosis. However, there are also some other advantages of AFM, such as the mechanical, electrical, or even chemical modes of AFM that may allow the exploration of some mechanical, electrical, or chemical information of the cell samples. As cell apoptosis might be associated with changes in cell membrane components, such as lipid rafts, membrane proteins, and phospholipids, there must be some mechanical, electrical, or even chemical changes on cell surface, which requires furthermore AFM investigations in our future works.

The theoretical resolution of AFM is much higher than optical microscope's resolution limit, which therefore allows the molecular or even atomic level imaging of samples. For cell sample imaging, AFM can provide the nanoscale properties of bacteria cells based on the high stiffness of bacteria surface [15], which proved their advantages for bacteria surface imaging than the optical microscope. However, the high-resolution imaging of living mammal cells is still restricted by the soft property of mammal cells. Here, we used AFM to image the fixed cancer cells, which could provide the accurate morphology of cancer cells with or without drug treatment. Optical microscopes allow very fast imaging of cell samples, which cannot be achieved by this AFM imaging technique due to its long duration of imaging times. Thus, for large scale and fast imaging of cell samples, an optical microscope is a better choice than AFM as it can also provide the information, such as shrink of cell bodies, condensation of cytoplasm, condensation, and fragmentation of nucleus. However, for the surface ultrastructure imaging, optical microscopy is largely restricted by their resolution limit in morphology imaging, whereas AFM could provide the precise structures with much higher resolution.

Up to now, the high-resolution imaging of living mammal cells is still very difficult due to the soft properties of their cell surface. There are still some restrictions to develop living mammal cell measurements of AFM into a quantitative and qualitative method for cell apoptosis detection that could be used by nonspecialists of AFM. For example, high-resolution living mammal cell membrane ultrastructure imaging by AFM (at a small area, such as 1 *μ*m and 2 *μ*m, or even nanometer-sized areas on cell surface) is still very difficult to be obtained, the influences of AFM tip on the growth situation of living cells during AFM measurements, and the limited time for AFM measurements by considering the influences of environments on living cells. Unlike living cell measurements of AFM, fixed cell imaging of AFM provides easier detection procedures and controllable detection time. For example, the high-resolution cell membrane ultrastructure can be easily detected by AFM after cell fixation; the cell samples after fixation can be stored at 4°C for further AFM imaging in one week. After fixation, the influences of AFM tip and the influences of environments on cell samples can be neglected because the cells are fixed. Therefore, fixed cells provide a more operability and simplification method for AFM measurements of cell samples, and that is why we used fixed cells for this study, which would be more helpful to extend this AFM method for ROS-dependent cancer cell apoptosis. However, the fixation processes and the following AFM imaging in air might introduce unexpected artifacts in cell samples. In fact, there are also some imaging techniques are widely used for high resolution cell imaging, such as scanning electron microscopy (SEM) and transmission electron microscopy (TEM). For SEM and TEM imaging, cells are also needed to be fixed and then be dehydrated for further sample preparation procedures and finally image the samples in vacuum, which are more harmful for cell samples. For AFM cell sample imaging in air, cell samples are always fixed and then dried in air; this is nondestructive compared with the cell sample preparation procedures of SEM and TEM. Thus, this cell sample preparation procedure for AFM imaging is widely used for high-resolution cell imaging through controlling the sample preparation procedures [36, 37].

In this work, we fixed the cells with 4% paraformaldehyde solution for 10 min, washed triple with distilled water, and dried in air overnight for AFM imaging in air. To extrude the effects of potential artifacts on the results of AFM imaging in different groups, all these operations were completed by the same person and the time was strictly controlled to make sure that every sample was treated at the same conditions. These criteria would be helpful to make sure that all the changes of cancer cells in this work were due to the drug treatment. We cannot deny the limitations of our current methodology that may introduce artifacts for the AFM imaging results, which did not provide the native property of living cells upon drug treatment. Although it is still a big challenge, we will try more works to achieve high-resolution imaging of living mammal cells in liquids in our following works, which might benefit the exploration drug effects in mammal cells.

## 4. Conclusions

In this study, we investigated the anticancer effects of GA on esophageal cancer EC9706 cells. Our results demonstrated that GA could inhibit cell proliferation, induce apoptosis, induce cell cycle arrest, and induce mitochondria membrane potential disruption in a ROS-dependent way, and the removal of GA-induced excessive ROS by NAC could reverse all these effects. Additionally, using AFM imaging, we also found that GA could induce the damage of morphology and membrane ultrastructure in EC9706 cells in a ROS-dependent way. GA was also found to increase the membrane height distribution and roughness of EC9706 cells in a ROS-dependent way, which could be also reversed by the removal of GA-induced intracellular ROS through NAC treatment. These results demonstrated the anticancer effects of GA on EC9706 cells in ROS-dependent mechanism and also strongly suggested AFM as a powerful tool for the study of ROS-mediated cancer cell apoptosis on the basis of imaging.

## Data Availability

The data that support the findings of this study are available on request from the corresponding authors.

## Supplementary Materials

Supplementary Materials Figure 1: effects of gambogic acid on cell morphology of EC9706 cells after 24 h treatment. Supplementary materials Figure 2: effects of gambogic acid on cell membrane height distribution of EC9706 cells after 24 h treatment.
