# Supplementary material for: Nanoscale Features of Gambogic Acid Induced ROS-Dependent Apoptosis in Esophageal Cancer Cells Imaged by Atomic Force Microscopy
Source: Scanning. 2022 Jul 22;2022:1422185. doi: 10.1155/2022/1422185 (PMC9337977; doi:10.1155/2022/1422185)
Supplement: Supplementary Materials — Figure 1: effects of gambogic acid on cell morphology of EC9706 cells after 24 h treatment. Supplementary materials Figure 2: effects of gambogic acid on cell membrane height distribution of EC9706 cells after 24 h treatment. [file 1422185.f1.docx]

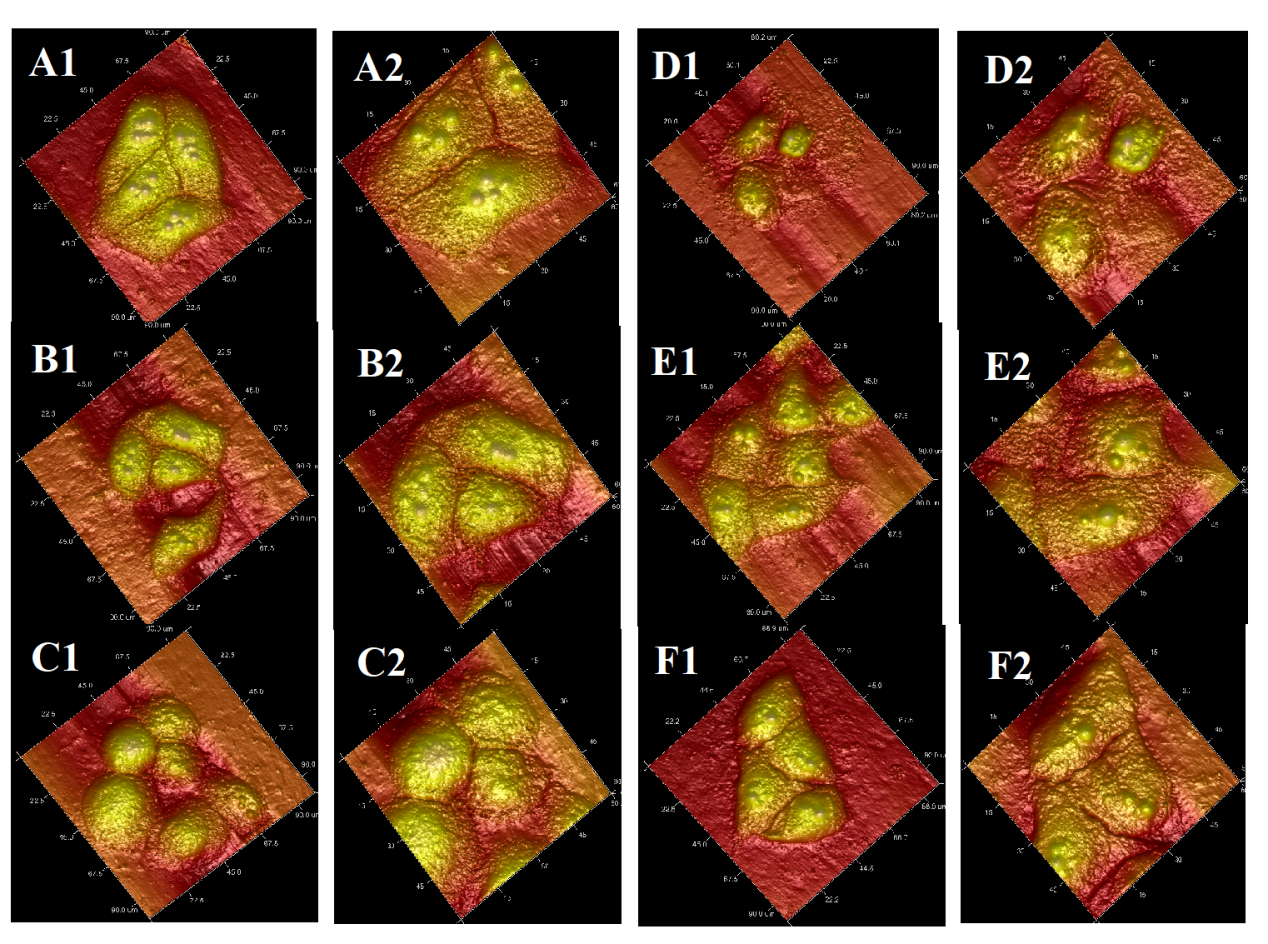
Supplementary materials Fig.1 Effects of gambogic acid on cell morphology of EC9706 cells after 24 h treatment. 3D images of (A) control, (B) 0.4 μM gambogic acid treated, (C) 1.2 μM gambogic acid treated, (D) 2 μM gambogic acid treated, (E) 5 mM NAC+2 μM gambogic acid treated and (F) 5mM NAC treated EC9706 cells obtained by AFM imaging corresponding to Fig.7. (A1-F1) 3D images of the topography from Fig.7A1-F1. (A2-F2) 3D images of the topography from Fig.7A3-F3.


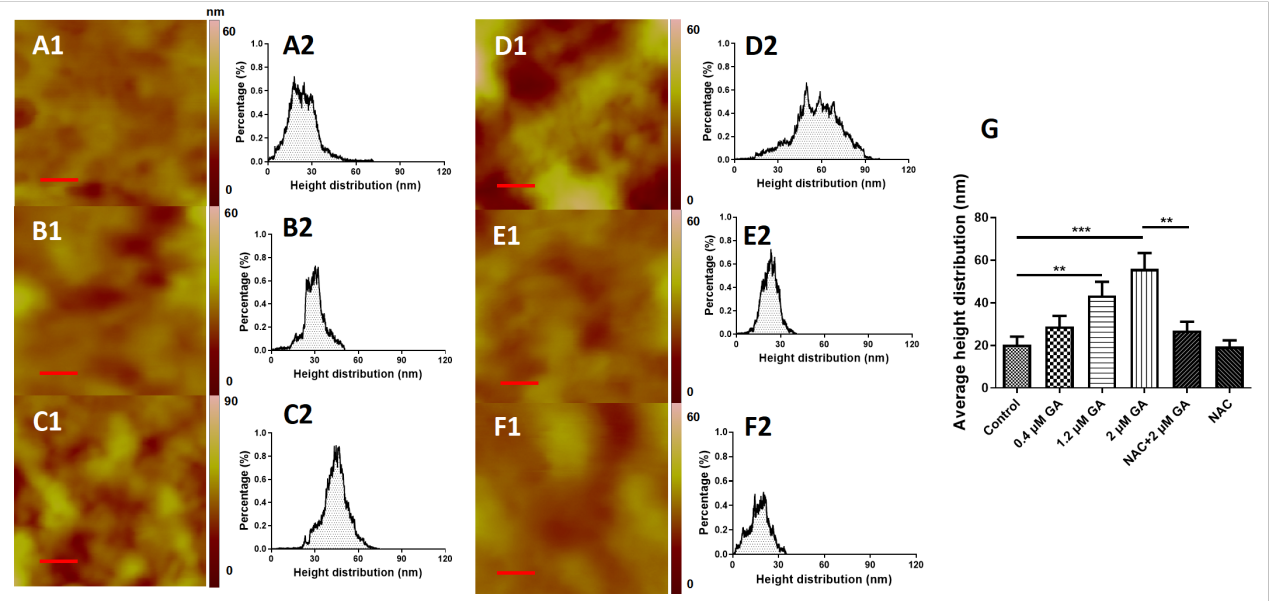


Supplementary materials Fig.2 Effects of gambogic acid on cell membrane height distribution distribution of EC9706 cells after 24 h treatment. AFM imaging of membrane ultrastructure images for (A) control, (B) 0.4 μM gambogic acid treated, (C) 1.2 μM gambogic acid treated, (D) 2 μM gambogic acid treated, (E) 5 mM NAC+2 μM gambogic acid treated and (F) 5mM NAC treated EC9706 cells. (A1-F1) Topography images and (A2-F2) their corresponding height distribution distribution in EC9706 cells, scale bar: 400 nm. (G) Height distribution analyzed from 2×2 μm frame ultrastructure images of EC9706 cells, n = 10, **p<0.01, ***p<0.001. EC9706 cells in (E) and (F) were pretreated with 5 mM NAC for 1 h and then treated with or without gambogic acid for 24 h to determine the effects of ROS scavenger-NAC on gambogic acid induced membrane ultrastructure changes in EC9706 cells.
